# Outcomes in Critically Ill Patients with Cancer-Related Complications

**DOI:** 10.1371/journal.pone.0164537

**Published:** 2016-10-20

**Authors:** Viviane B. L. Torres, Juliana Vassalo, Ulysses V. A. Silva, Pedro Caruso, André P. Torelly, Eliezer Silva, José M. M. Teles, Marcos Knibel, Ederlon Rezende, José J. S. Netto, Claudio Piras, Luciano C. P. Azevedo, Fernando A. Bozza, Nelson Spector, Jorge I. F. Salluh, Marcio Soares

**Affiliations:** 1 Postgraduate Program in Internal Medicine, School of Medicine, Universidade Federal do Rio de Janeiro, Rio de Janeiro, RJ, Brazil; 2 ICU, Fundação Pio XII, Hospital do Câncer de Barretos, Barretos, Brazil; 3 ICU, A.C. Camargo Cancer Center, São Paulo, Brazil; 4 Rede Institucional de Pesquisa e Inovação em Medicina Intensiva (RIPIMI), Irmandade da Santa Casa de Misericórdia de Porto Alegre, Porto Alegre, Brazil; 5 ICU, Hospital Israelita Albert Einstein, São Paulo, Brazil; 6 ICU, Hospital Português, Salvador, Brazil; 7 Hospital São Lucas, Travessa Frederico Pamplona 32, Rio de Janeiro, Brazil; 8 ICU, Hospital do Servidor Público Estadual, São Paulo, Brazil; 9 ICU, Instituto Nacional de Câncer, Hospital do Câncer II, Rio de Janeiro, Brazil; 10 ICU, Vitória Apart Hospital, Vitória, Brazil; 11 Research and Education Institute, Hospital Sírio-Libanês, São Paulo, Brazil; 12 IDOR, D’Or Institute for Research and Education, Rio de Janeiro, Brazil; 13 National Institute of Infectious Disease Evandro Chagas, Oswaldo Cruz Foundation (FIOCRUZ), Rio de Janeiro, Brazil; 14 Postgraduate Program, Instituto Nacional de Câncer, Rio de Janeiro, Brazil; Fundacao Oswaldo Cruz, BRAZIL

## Abstract

**Introduction:**

Cancer patients are at risk for severe complications related to the underlying malignancy or its treatment and, therefore, usually require admission to intensive care units (ICU). Here, we evaluated the clinical characteristics and outcomes in this subgroup of patients.

**Materials and Methods:**

Secondary analysis of two prospective cohorts of cancer patients admitted to ICUs. We used multivariable logistic regression to identify variables associated with hospital mortality.

**Results:**

Out of 2,028 patients, 456 (23%) had cancer-related complications. Compared to those without cancer-related complications, they more frequently had worse performance status (PS) (57% vs 36% with PS≥2), active malignancy (95% vs 58%), need for vasopressors (45% vs 34%), mechanical ventilation (70% vs 51%) and dialysis (12% vs 8%) (*P*<0.001 for all analyses). ICU (47% vs. 27%) and hospital (63% vs. 38%) mortality rates were also higher in patients with cancer-related complications (*P*<0.001). Chemo/radiation therapy-induced toxicity (6%), venous thromboembolism (5%), respiratory failure (4%), gastrointestinal involvement (3%) and vena cava syndrome (VCS) (2%) were the most frequent cancer-related complications. In multivariable analysis, the presence of cancer-related complications per se was not associated with mortality [odds ratio (OR) = 1.25 (95% confidence interval, 0.94–1.66), *P* = 0.131]. However, among the individual cancer-related complications, VCS [OR = 3.79 (1.11–12.92), *P* = 0.033], gastrointestinal involvement [OR = 3.05 (1.57–5.91), *P* = <0.001] and respiratory failure [OR = 1.96(1.04–3.71), *P* = 0.038] were independently associated with in-hospital mortality.

**Conclusions:**

The prognostic impact of cancer-related complications was variable. Although some complications were associated with worse outcomes, the presence of an acute cancer-related complication *per se* should not guide decisions to admit a patient to ICU.

## Introduction

The number of patients with malignancies admitted to intensive care units (ICU) has increased over the last decades, and outcomes seem to be improving in several subsets of patients [1–5]. Although triage decisions based solely on the underlying malignancy are no longer supported, a diagnosis of cancer is still one of the main reasons for refusal of admission to the ICU [6, 7]. In order to assist clinical decisions, recent studies have identified important determinants of mortality, such as severity of acute organ failures and performance status (PS), and have conversely cast doubt on other traditional predictors as neutropenia and autologous bone-marrow transplant [8–11]. However, as cancer is an heterogeneous and complex disease, the identification of those who are most likely to benefit from intensive care remains a challenge, in order to guide triage decisions and avoid inappropriate care in patients with a poor life expectancy [12, 13].

Acute complications related to cancer or its treatment are often the reason for ICU admission. Complications can arise as the initial manifestation of a malignancy or due to its progress, and require urgent therapeutic interventions [14, 15]. A better understanding of such complications and their impact on patients’ outcomes is essential to optimize care planning, use of ICU resources, and for the counseling of relatives and patients [16]. However, to our knowledge, the existing literature is scarce and usually limited to specific subgroups of patients and complications [17–20]. In the present study, we evaluated the clinical characteristics and outcomes in patients admitted to ICUs with complications related to cancer or its treatment. We also assessed the impact of these complications on the hospital mortality.

## Materials and Methods

### Design, Setting and Eligibility Criteria

In this study, we performed an analysis of two prospective cohort studies in critically ill cancer patients: study 1—a single center study performed from January 2003 to July 2007 at the Instituto Nacional do Câncer (INCA), Rio de Janeiro, Brazil; and study 2—a multicenter study conducted in 28 Brazilian ICUs between August 1^st^ and September 30^th^, 2007 [21]. The studies were observational and did not interfere with routine medical practice. The study 1 was approved by the Ethics Committee (EC) of the Instituto Nacional de Câncer (INCA) (approval numbers 12/2001 and 10/2003). The study 2 was initially approved by the EC at Instituto Nacional do Câncer, the coordinating center, (approval number 013/07) and subsequently by the Brazilian National EC (CONEP, approval number 13.914). Following the approval by the CONEP, the last study was therefore approved by local ECs at each participating centers. The need for informed consent was waived in both studies. Eligibility criteria, data collection and processing as well as variables definitions were equivalent in both studies.

We evaluated all adult patients (≥18 years) with a definite cancer diagnosis admitted to the participating ICUs. We excluded patients in complete cancer remission for more than five years, those with an ICU stay of less than 24 hours and readmissions.

### Data Collection and Definitions

We collected the following information in every patient studied: demographics, clinical and laboratory data including comorbidities, ICU admission diagnoses, the type of admission (medical or surgical), the Sequential Organ Failure Assessment (SOFA) score [22] and the second version of the Simplified Acute Physiology Score (SAPS 2) [23]. Comorbidities were assessed according to the Adult Comorbidity Evaluation—27 (ACE-27), which grades a wide range of comorbid diseases and conditions according to the severity of organ decompensation and prognostic impact. An overall comorbidity score (none, mild, moderate, or severe) is assigned based on the highest-ranked single ailment [24]. We defined organ failure as a SOFA score ≥2 points for the organ in question [25] and assessed the need for dialysis, vasopressors and ventilatory support (invasive and non-invasive mechanical ventilation) at admission and during ICU stay. Cancer- and treatment-related variables were also recorded and this included performance status (PS) [Eastern Cooperative Oncology Group scale] [26] and type of cancer (solid or hematological malignancy). For solid tumors, we recorded the presence of metastases. The type of hematologic malignancy was categorized into high-grade malignancies, including acute myelogenous leukemia, acute lymphoblastic leukemia, and high-grade non-Hodgkin lymphoma, and into low-grade malignancies, including all other types of hematologic malignancies and aplastic anemia [27, 28].

In the two cohorts, all patients were assessed for the presence of acute complications related to the underlying malignancy or its treatment at ICU admission, comprising: chemo- and radiation therapy toxicities (hematologic, mucositis and others, which included cardiac and pulmonary adverse events; according to the Common Terminology Criteria for Adverse Events) [29], venous thromboembolism [pulmonary embolism and/or deep vein thrombosis (DVT) confirmed by clinical examination and appropriate imaging methods] [30], respiratory failure by tumor (mechanical obstruction, lung infiltration and/or lymphangitic carcinomatosis, stated by pre-defined criteria) [31], central neurological complications related to tumor (brain mass effect, spinal cord compression syndrome and seizure/status epilepticus) [32], neutropenia due to bone marrow infiltration by tumor, vena cava syndrome (VCS) (established by clinical and imaging methods) [33], acute tumor lysis syndrome (established by defined criteria) [34], malignant massive pleural and pericardial effusion (diagnosed by fluid cytology and imaging methods) [35, 36], hypercalcemia (defined as a serum calcium level> 10.2 mg / dL or ionized calcium> 1.23 mmol / L) associated with malignancy [37] and urinary tract obstruction or infiltration by tumor. Gastrointestinal complications by tumor meet the following criteria definition: perforation and / or bleeding that occurred at the primary site of the tumor and gastrointestinal or biliary obstruction caused by intraluminal tumor or extrinsic compression [14]. Other complications such as hyperviscosity syndrome, multiple myeloma- related kidney injury, bleeding secondary to dyscrasia were also evaluated and grouped in the same variable for analysis.

We defined infection as the presence of a pathogenic microorganism in a sterile site (such as blood, cerebrospinal fluid or ascites) or clinically suspected infection that needed administration of antibiotics [3, 38]. Sepsis was defined according to current consensus definitions [39]. Cancer status was classified as "in remission/ controlled" (ie, patients in cancer remission or control who have undergone previous treatments, without evidence of recurrence according to the attending oncologist/ hematologist), "active—newly diagnosed" (diagnosed within the last 3 months) and "active—relapse/progression" (recurrent disease). Vital status at hospital discharge was the main outcome of interest.

### Statistical Analysis

We performed statistical analyses using SPSS 21.0 for Windows (SPSS Inc., Chicago, IL, USA). We reported discrete variables as counts (percentage) and continuous variables as mean±standard deviation or median (25%-75% interquartile range, IQR) as appropriate. Data completeness was good with very few missing data for the length of stay in ICU (n = 2) and hospital (n = 9). In these cases, we used simple imputation using median values. For demographics and clinical characteristics of the study groups, we assessed differences between groups using the chi-square test, Fisher's exact test, Student's t-test or Mann-Whitney U test, as appropriate. We used univariate and multivariable logistic regression analyses to identify factors associated with hospital mortality [40]. Variables yielding *P* values <0.2 by univariate analysis and those considered clinically relevant were entered in the multivariable analysis to estimate the independent association of each covariate with the dependent variable. We performed three models of multivariable logistic regression analyses. In the first analysis, the relation between mortality and the presence of a cancer-related complication (yes or no) was tested. In the second model, the association between mortality and the number (one or more than one) of complications presented by the patient was evaluated. Finally, the prognostic impact for the individual complications was investigated. Results of multivariable analysis were summarized as odds-ratios (OR) and respective 95% intervals (CI). Possible interactions were tested. The model’s calibration was assessed using the Hosmer-Lemeshow goodness-of-fit test [40]. With this test, *P* values >0.05 indicate a good fit for the model. We considered a two-tailed *P*<0.05 as significant for all other statistics.

## Results

### Characteristics of the Study Population

A total of 2,028 patients (study 1, n = 1,311; study 2, n = 717) admitted to the participating ICUs were eligible during the study period and formed the study population. **Table 1** depicts the patients' main characteristics.

**Table 1:** Main patients’ characteristics and univariate analysis for in-hospital mortality.^a^

Variables	All patients (*N* = 2028)	Survivors (*N* = 1141, 56%)	Non-survivors (*N* = 887, 44%)	Odds-ratio (95% CI)	*P* Value ^b^	
**Age (years)**	62 (50–71)	60 (49–70)	63 (51–72)	1.01 (1.00–1.01)	0.008	
**Male gender**	1072 (53%)	579 (51%)	493 (56%)	1.26 (1.02–1.45)	0.030	
**Hospital stay before ICU admission (days)**	2 (1–7)	2 (1–6)	3 (1–8)	1.03 (1.02–1.04)	<0.001	
**Medical admission**	1028 (51%)	400 (35%)	628 (71%)	4.49 (3.72–5.43)	<0.001	
**Comorbidity score (ACE-27)**						
None-mild	1412 (70%)	809 (71%)	603 (68%)	1.00	0.156	
Moderate-severe	616 (30%)	332 (29%)	284 (32%)	1.15 (0.95–1.39)		
**Type of cancer**						
Locoregional solid tumor	1331 (66%)	862 (76%)	469 (53%)	1.00	<0.001	
Metastatic solid tumor	406 (20%)	183 (16%)	223 (25%)	2.24 (1.79–2.81)	<0.001	
Hematological malignancy (low grade)	106 (5%)	44 (4%)	62 (7%)	2.59 (1.73–3.87)	<0.001	
Hematological malignancy (high grade)	185 (9%)	52 (5%)	133 (15%)	4.70 (3.35–6.60)	<0.001	
**Cancer status**						
Controlled / remission	690 (34%)	437 (38%)	253 (29%)	1.00	<0.001	
Active—newly-diagnosed	830 (41%)	468 (41%)	362 (41%)	1.34 (1.09–1.64)	0.006	
Active—recurrence / progression	508 (25%)	236 (21%)	272 (31%)	1.99 (1.58–2.51)	<0.001	
**Performance status**						
0–1	1210 (60%)	848 (74%)	362 (41%)	1.00	<0.001	
2–4	818 (40%)	293 (26%)	525 (59%)	4.20 (3.48–5.07)		
**Previous chemotherapy**	664 (33%)	333 (29%)	331 (37%)	1.45 (1.19–1.74)	<0.001	
**Previous radiotherapy**	510 (25%)	261 (23%)	249 (28%)	1.32 (1.08–1.61)	0.009	
**Severity of acute illness at ICU admission**							
SAPS II score (points)	39 (26–53)	30 (21–40)	51 (41–64)	1.09 (1.08–1.09)		<0.001	
SOFA on the 1^st^ day of ICU (points)	7 (5–10)	6 (4–8)	9 (6–12)	1.26 (1.23–1.30)		<0.001	
Infection	920 (45%)	343 (30%)	577 (65%)	4.33 (3.59–5.22)		<0.001	
Mechanical ventilation	1119 (55%)	421 (37%)	698 (79%)	6.32 (5.17–7.72)		<0.001	
Vasopressors	733 (36%)	236 (21%)	497 (56%)	4.89 (4.02–5.94)		<0.001	
Dialysis	175 (9%)	59 (5%)	116 (13%)	2.76 (1.99–3.83)		<0.001	
**Events during ICU stay**							
Infection	281 (14%)	102 (9%)	179 (20%)	2.58 (1.98–3.34)		<0.001	
Mechanical ventilation	1,291 (64%)	507 (44%)	784 (88%)	9.52 (7.52–12.06)		<0.001	
Vasopressors	981 (48%)	313 (27%)	668 (75%)	8.07 (6.60–9.86)		<0.001	
Dialysis	315 (16%)	77 (7%)	238 (27%)	5.09 (3.87–6.70)		<0.001	
ICU chemotherapy/ radiotherapy	71 (4%)	25 (2%)	46 (5%)	2.44 (1.49–4.01)		<0.001	
**Outcomes data**							
End-of-life decisions	339 (17%)	7 (1%)	332 (37%)	-		<0.001	
ICU LOS (days)	5 (3–12)	4 (2–9)	7 (3–15)	1.02 (1.02–1.03)		<0.001	
Hospital LOS (days)	17 (9–34)	18 (9–34)	17 (8–33)	0.99 (0.99–1.00)		0.645	

^a^ Results expressed as mean±SD, median (25%-75% IQR), n (%); IQR = interquartile range

^b^ Reported *P* values refer to comparisons between survivors and non-survivors

ACE-27 = Adult Comorbidity Evaluation; ICU = Intensive Care Unit; LOS = length of stay; SAPS = Simplified Acute Physiology Score; SOFA = Sequential Organ Failure Assessment.

There were 1,737 (86%) patients with solid tumors and 291 (14%) patients with hematological malignancies (**Table 1**). The most common primary sites of solid tumor were gastrointestinal tract (26%), head and neck (11%), brain (10%), lung (9%), urological (9%) and breast (6%). The main hematological malignancies were non-Hodgkin’s lymphomas (7%), acute leukemias (3%), multiple myeloma (2%) and Hodgkin’s lymphoma (1.4%). A total of 456 (23%) had cancer-related complications at ICU admission. The most frequent complications were chemo- and radiation therapy toxicity (26%), venous thromboembolism (21%) and respiratory failure by tumor (17%) **(Table 2)**.

**Table 2:** The main cancer related-complications and univariate analysis of predictors of hospital mortality.^a^

Variables	All patients (*N* = 2,028)	Survivors (*N* = 1,141, 56%)	Non-survivors (*N* = 887, 44%)	Odds-ratio (95% CI)	*P* Value ^b^
**Presence of Complications**					
No	1572 (78%)	970 (85%)	602 (68%)	1.00	<0.001
One complication	352 (17%)	138 (12%)	214 (24%)	2.49 (1.97–3.17)	
≥ 2 complications	104 (5%)	33 (3%)	71 (8%)	3.47 (2.27–5.30)	
**Type of Complication**					
Chemotherapy / radiotherapy toxicity	117 (6%)	50 (4%)	67 (8%)	1.78 (1.22–2.60)	0.002
Chemotherapy toxicity	107 (5%)	44 (4%)	63 (7%)	1.9 (1.28–2.83)	0.001
*Hematologic*	85 (4%)	28 (3%)	57 (6%)	2.73 (1.72–4.33)	<0.001
*Mucositis*	16 (1%)	5 (0.5%)	11 (1%)	2.85 (0.99–8.24)	0.073
*Others*	21 (1%)	16 (1%)	5 (1%)	-	-
Radiotherapy toxicity	12 (1%)	7 (1%)	5 (1%)	0.92 (0.29–2.90)	0.999
Pulmonary embolism / DVT	96 (5%)	46 (4%)	50 (6%)	1.42 (0.94–2.14)	0.091
Respiratory failure by tumor	77 (4%)	17 (2%)	60 (7%)	4.79 (2.78–8.28)	<0.001
Central neurological complications	54 (3%)	22 (2%)	32 (4%)	1.90 (1.10–3.30)	0.020
*Spinal cord compression syndrome*	13 (1%)	5 (0.5%)	8 (1%)	2.07 (0.67–6.34)	0.263
*Brain mass effect*	42 (2%)	17 (1%)	25 (3%)	1.91 (1.03–3.57)	0.037
*Seizure/ status epilepticus*	9 (0.5%)	5 (0.5%)	4 (0.5%)	1.03 (0.28–3.84)	0.966
GI tract complications	59 (3%)	16 (1%)	43 (5%)	3.58 (2.00–6.40)	<0.001
*GI perforation/obstruction / bleeding*	49 (2%)	16 (1%)	33 (4%)	2.71 (1.49–4.97)	0.001
*Biliary obstruction*	10 (0.5%)	0 (0%)	10 (1%)	-	<0.001
Neutropenia	35 (2%)	12 (1%)	23 (3%)	2.50 (1.24–5.06)	0.010
Vena cava syndrome	32 (2%)	4 (0.5%)	28 (3%)	9.27 (3.24–26.5)	<0.001
Tumor lysis syndrome	24 (1%)	8 (1%)	16 (2%)	2.60 (1.11–6.11)	0.036
Pleural/ pericardial effusion	20 (1%)	7 (1%)	13 (2%)	2.41 (0.96–6.07)	0.069
Urinary tract obstruction	17 (1%)	10 (1%)	7 (1%)	0.90 (0.34–2.34)	0.999
Hypercalcemia	16 (1%)	4 (0.5%)	12 (1%)	3.89 (1.25–12.1)	0.020
Others	33 (2%)	15 (1%)	18 (2%)	1.56 (0.78–3.10)	0.207

^a^ Results expressed as n (%).

^b^ Reported *P* values refer to comparisons between survivors and non-survivors

CI = confidence interval; DVT = deep venous thrombosis; GI = gastrointestinal.

### Comparisons Between Patients With and Without Cancer-related Complications

We reported 456 patients who had a complication related to cancer at ICU admission as a primary cause of admission or as a factor that contributed to the acute condition. Comparisons between patients with and without cancer complications are in **Table 3**. Patients with complications were younger [59 (45–69) years vs. 62 (51–72) years, *P*<0.001], had more frequently worse PS (57% vs. 36% with PS ≥ 2, *P*<0.001) and active disease (95% vs. 58%, *P*<0.001). They also presented with higher severity of organ dysfunctions, need for invasive support and infection at ICU admission (**Table 3**).

**Table 3:** Main patients’ characteristics and comparisons between patients with and without cancer-related complications.^a^

Variables	*All patients (N = 2*,*028)*	Patients with complications (*N* = 456, 23%)	Patients without complications (*N* = 1,572, 77%)	*P* value ^b^
**Age (years)**	62 (50–71)	59 (45–69)	62 (51–72)	<0.001
**Male gender**	1072 (53%)	249 (55%)	823 (52%)	0.427
**Comorbidity score (ACE-27)**				0.487
None-mild	1412 (70%)	324 (71%)	1088 (69%)	
Moderate-severe	616 (30%)	132 (29%)	484 (31%)	
**Type of admission**				<0.001
Medical admission	1028 (51%)	389 (85%)	639 (41%)	
Schedule surgery	654 (32%)	3 (1%)	651 (41%)	
Emergency surgery	346 (17%)	64 (14%)	282 (18%)	
**Hospital days prior to ICU admission**	2 (1–7)	3 (0–8)	2 (1–7)	0.308
**Type of cancer**				
Locoregional solid tumor	1331 (66%)	198 (43%)	1133 (72%)	<0.001
Metastatic solid tumor	406 (20%)	113 (25%)	293 (19%)	
Hematological malignancy (low grade)	106 (5%)	32 (7%)	74 (5%)	
Hematological malignancy (high grade)	185 (9%)	113 (25%)	72 (5%)	
**Cancer status**				
Controlled / remission	690 (34%)	21 (5%)	669 (42%)	<0.001
Active cancer—newly-diagnosed	830 (41%)	251 (55%)	579 (37%)	
Active cancer—recurrence / progression	508 (25%)	184 (40%)	324 (21%)	
**Anticancer treatments prior to ICU admission**				
Chemotherapy	664 (33%)	210 (46%)	454 (29%)	<0.001
Radiation therapy	510 (25%)	128 (28%)	382 (25%)	0.123
**Performance status**				
0–1	1210 (60%)	197 (43%)	1013 (64%)	<0.001
2–4	818 (40%)	259 (57%)	559 (36%)	
**Severity of acute illness at ICU admission**				
SAPS II score–admission (points)	39 (26–53)	49 (38–60)	35 (24–50)	<0.001
SOFA on the first day of ICU (points)	7 (5–10)	8 (5–11)	6 (4–9)	<0.001
Infection	920 (45%)	260 (57%)	660 (42%)	<0.001
Mechanical ventilation	1119 (55%)	320 (70%)	799 (51%)	<0.001
Vasopressors	733 (36%)	206 (45%)	527 (34%)	<0.001
Acute renal injury	459 (23%)	146 (32%)	313 (20%)	<0.001
Dialysis	175 (9%)	56 (12%)	119 (8%)	0.002
**Events during ICU stay**				
Infection	281 (14%)	68 (15%)	213 (13%)	0.063
Mechanical ventilation	1291 (64%)	346 (76%)	945 (60%)	<0.001
Vasopressors	981 (48%)	275 (60%)	706 (45%)	<0.001
Dialysis	315 (16%)	92 (20%)	223 (14%)	0.002
**Outcomes data**				
End-of-life decisions	339 (17%)	133 (29%)	206 (13%)	<0.001
ICU LOS (days)	5 (3–12)	6 (3–13)	5 (3–12)	0.003
Hospital LOS (days)	17 (9–34)	19 (10–33)	17 (9–34)	0.501
ICU mortality	643 (32%)	216 (47%)	427 (27%)	<0.001
Hospital mortality	887 (44%)	285 (63%)	602 (38%)	<0.001

^a^ Results expressed as mean±SD, median (25%-75% IQR), n (%); IQR = interquartile range

^b^ Reported *P* values refer to comparisons between patients with or without cancer-related complications

ACE-27 = Adult Comorbidity Evaluation; ICU = Intensive Care Unit; LOS = length of stay; SAPS = Simplified Acute Physiology Score; SOFA = Sequential Organ Failure Assessment.

Complications were more frequent in patients with metastatic solid tumors and those with more aggressive hematologic malignancies. Among patients with solid tumors, complications were more common in lung and breast cancers, and conversely, less frequent in patients with gastrointestinal tumors (data not shown). Among hematologic patients, complications were more frequent in those with acute leukemias and aggressive non-Hodgkin’s lymphoma.

### Outcome Data

The main patients’ characteristics and comparisons between survivors and non-survivors are shown in **Table 1**. In univariate analyses, the presence of chemotherapy-induced hematologic toxicities, neutropenia by tumor, acute tumor lysis syndrome, hypercalcemia by tumor, central neurological complications, vena cava syndrome (VCS), respiratory failure by tumor and gastrointestinal tract complications were associated with increased mortality. Of note, all patients with tumoral biliary obstruction died (**Table 2)**.

The overall ICU and hospital mortality were 32% (643) and 44% (887), respectively. Median ICU and hospital lengths of stay (LOS) were 5 (3–12) and 17 (9–34) days, respectively. Compared to patients without cancer-related complications, those with cancer-related complications had higher ICU and hospital mortality rates (47% vs. 27% and 63% vs. 38%, *P*<0.001, respectively), as well as, higher ICU LOS [6 (3–13) vs. 5 (3–12) days, *P* = 0.003]. Out of 456, 133 (29%) patients with cancer-related complications had end-of-life decisions (in general, to withhold life-sustaining treatments) at ICU compared to 13% of patients without cancer-related complications (*P* = 0.003) (**Table 3**). Thirty-nine (9%) patients with cancer-related complications received urgent chemo- and/or radiation therapy during ICU stay and 26 (67%) of them died in the hospital.

We performed multivariable analyses to investigate whether the presence of cancer-related complications at ICU admission were associated with hospital mortality adjusting for age, gender, hospital LOS before ICU admission, type of admission (medical or surgical) comorbidities (ACE-27 score none-mild or moderate-severe), cancer type (locoregional solid tumor, metastatic solid tumor, low grade hematological malignancy or high grade hematological malignancy) and status (controlled / remission, active cancer newly-diagnosed or active cancer in recurrence or progression), PS (0–1 or 2–4), previous anticancer treatments (chemotherapy or radiation therapy), SOFA score and use of mechanical ventilation on the first ICU day. In the first analysis, the presence of cancer-related complications *per se* (yes or no) [OR = 1.25 (95% CI, 0.94–1.66), *P* = 0.131] was not independently associated with mortality. In the second model, we also did not find association between the number of cancer-related complications presented by the patient and hospital outcome for one complication [OR = 1.22 (0.90–1.66), *P* = 0.198], and when two or more complications [OR = 1.36 (0.80–2.29), *P* = 0.254] were present. Subsequently, we investigated the prognostic impact for the individual complications. In this analysis, cancer-related VCS [OR = 3.79 (1.11–12.92)], gastrointestinal complications [OR = 3.05 (1.57–5.91)] and respiratory failure [OR = 1.96 (1.04–3.71)] were associated with increased mortality (**Table 4**).

**Table 4:** Multivariable analysis of variables associated with hospital mortality.

Variables	Coefficients	OR (95% CI)	*P* value
**Age (years)**	0.017	1.02 (1.01–1.02)	<0.001
**Gender**			
Female		1.00	
Male	0.192	1.21 (0.97–1.51)	0.086
**Hospital days prior to ICU admission**	0.105	1.11 (1.02–1.21)	0.019
**Type of admission**			
Surgical		1.00	
Medical	0.691	1.99 (1.58–2.53)	<0.001
**Type of cancer**			
Locoregional solid tumor		1.00	
Metastatic solid tumor	0.755	2.12 (1.60–2.83)	<0.001
Hematological malignancy (low grade)	0.347	1.42 (0.88–2.27)	0.151
Hematological malignancy (high grade)	0.749	2.12 (1.39–3.21)	<0.001
**Performance status** ^**a**^			
0–1		1.00	
2–4	0.895	2.45 (1.96–3.07)	<0.001
**SOFA score at ICU admission (points)**	0.155	1.17 (1.13–1.21)	<0.001
**Mechanical ventilation at ICU admission**	1.349	3.85 (3.05–4.88)	<0.001
**Respiratory failure by tumor**	0.673	1.96 (1.04–3.71)	0.038
**GI tract complications** ^**a**^	1.114	3.05 (1.57–5.91)	<0.001
**Vena cava syndrome**	1.333	3.79 (1.11–12.9)	0.033
**Constant**	-4.476		

N = 2,028. Hosmer-Lemeshow goodness-of-fit (χ2 = 7.270, *P* = 0.508). CI = confidence interval, ICU = Intensive care unit; GI = gastrointestinal; OR = odds ratio; SOFA = Sequential Organ Failure Assessment.

^a^ For these variables, the OR are also affected by the associated interaction term: GI tract complications and performance status ≥ 2

[coefficient -1.719; OR = 0.18 (0.48–0.67); *P* = 0.011].

**Fig 1:**
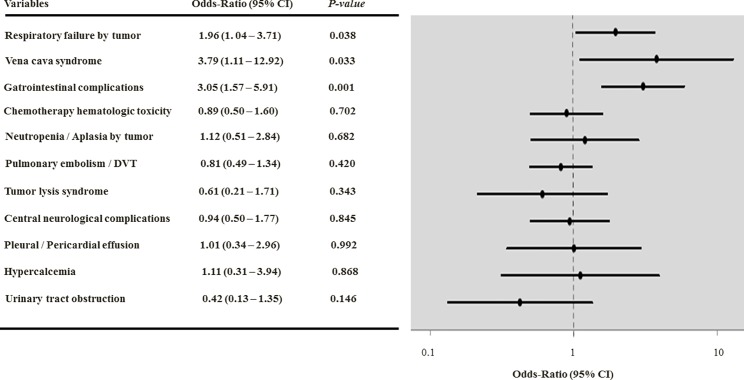
Multivariable analysis and adjusted odds ratios for hospital mortality of critically ill patients with cancer-related complications. Odds ratios greater than 1.0 indicate an increased risk of death. Constant: -4.476. Hosmer-Lemeshow goodness-of-fit (*χ*^2^ = 7.270; *P* = 0.508). CI = confidence interval.

We observed interaction between the PS and gastrointestinal complications. The prognostic impact of each complication is depicted in **Fig 1**.

## Discussion

In the present study, we performed a detailed assessment of cancer-related complications in in critically ill patients. Our study has three major findings: 1) approximately one in four patients with cancer admitted to ICUs presented with acute complications related to the underlying malignancy or its treatment's side effects; 2) there are many cancer-related complications, and their prognostic impact is quite variable; and 3) despite high mortality rates, outcome in these patients is better than perceived *a priori*. Among the complications studied, only VCS, gastrointestinal involvement and respiratory failure were independently associated with in-hospital mortality, probably due to the lack of specific treatments, and because they are manifestations of advanced and uncontrolled cancer. Close collaboration among onco-hematologists and intensivists has been recommended by experts as paramount to achieving optimal care of cancer patients requiring ICU admission [41]. These patients reap the greatest benefit from such collaborative management. Our results can be of help in assisting in decisions related to patients’ care, including triage for ICU admission, decisions to offer urgent anticancer treatments and in patient and family counseling.

Previous studies that evaluated specific malignancies in the intensive care setting observed mortality rates varying according to the nature of the complication, however most often; this condition has been implicated with worse outcomes [17–20]. One of the main complications that have been reported as a predictor of poor survival, in congruence with our findings, is the pulmonary/airway involvement by the cancer as the reason to the respiratory failure. It has been observed in several subsets of patients, such those requiring ventilatory support and with hematological malignancies [5, 10]. Soares et al also have confirmed the worse outcomes in the subgroup of critically ill patients with lung cancer [8]. Likewise, both airway compromise by tumor as superior vena cava syndrome were independent predictors for mortality in another multicenter study performed in pulmonary malignancies. The other risk factors for adverse outcomes found by these authors was DVT, in contrast with our findings [13]. Although venous thromboembolism was one of the most frequent complications in our cohort, it was not a major determinant of outcome. In accordance with our results, Valade et al evaluated severe pulmonary embolism and showed malignancy as a risk factor for life-threatening complications, like major bleeding and cardiac arrest, but not for death [42].

Another complication found in our study as an independent predictor of mortality was gastrointestinal involvement by tumor. Although the mortality in gastric/intestinal perforation and severe bleeding by tumor has been reported in different studies [43–46], data regarding the prognostic factors of this complication in the setting of critical care are scarce. The substantial mortality rate of 73% observed in our cohort emphasizes the importance of discussing the appropriateness of ICU admission in this specific subgroup of patients.

An important point of our study was the high frequency of chemo/radiation therapy-induced toxicity. Treatment-related neutropenia was not a good predictor of outcome, which is in congruence with previously published data, since it is no longer considered a relevant predictor of mortality [3, 11].

The other independent predictors of mortality we observed were high SOFA score performed on the first day of ICU stay, worse PS and need for mechanical ventilation. These findings are in agreement with the current literature, once patient level of functioning and the number of organ dysfunctions are the leading determinants of prognosis in critically ill patients with cancer [3, 10, 41, 47]. According to the knowledge on cancer patients, it is reasonable to hypothesize that prompt recognition and early intervention before the physiological derangement are important measures that may impact directly on mortality and morbidity of this high-risk subgroup [48, 49].

Our study has several strengths, but also has several limitations. First, we studied only patients admitted to ICUs presenting complications at admission. Therefore, our results do not represent the full spectrum of clinical presentation of acute cancer complications. Some subgroups, such as those with prolonged neutropenia and those with bone marrow transplant-related complications, are under-represented or were not evaluated. Besides, the clinical course of those whose ICU admission was refused, or who were not referred to the ICU, was not assessed and the patients admitted to the participating ICUs were, in most cases, considered suitable for referencing by oncologists and hematologists. Therefore, these results should be used with caution to a general patients with cancer-related complications. Second, survival rates in critically ill cancer patients do not depend only on patients’ characteristics. Organizational characteristics and processes of care of the medical centers that potentially affect patients’ outcomes were not assessed in our study. Third, information about long-term survival and disease-free survival are needed for a more appropriate assessment of patients’ outcomes, but were not available. Finally, only a few patients received urgent anticancer treatments in the ICU. However, even in specialized centers, such treatments are offered to very selected patients. Future studies are required to investigate the impact and appropriateness of urgent anticancer treatments in the ICU.

In conclusion, acute cancer-related complications are frequent reasons for ICU admission in onco-hematological patients. As the prognostic impact is variable, the presence of such complications *per se* should neither guide decisions to admit a patient to the ICU nor to limit life-sustaining therapies.

## Supporting Information

S1 File Demographics, clinical and laboratory patients data, cancer- and treatment-related variables, the acute complications related to the underlying malignancy or its treatment at ICU admission, characteristics of the acute illness at ICU admission, the events during ICU stay and outcomes data. (XLSX)
